# Developing European policy on xenotransplantation: instructive findings from the first representative UK sample survey on attitudes and views on animal to human transplants

**DOI:** 10.3389/ti.2026.16585

**Published:** 2026-09-15

**Authors:** Mustafa Al-Haboubi, Paul Boadu, Agata Pacho, Nicholas Mays, Antonia J. Cronin

**Affiliations:** 1 Policy Innovation and Evaluation Research Unit (PIRU), Department of Health Services Research and Policy, London School of Hygiene and Tropical Medicine, London, United Kingdom; 2 Guy’s and St. Thomas', Transplant Renal and Urology Directorate, 6th Floor Borough Wing, Guy’s Hospital, London, United Kingdom; 3 King’s College, London, School of Immunology and Microbial Science, Faculty of Life Sciences and Medicine, 5th Floor Tower Wing, Guy’s Hospital, London, United Kingdom

**Keywords:** European policy, health policy, organ donation, public attitudes, xenotransplantation

## Abstract

Recent landmark advances, particularly those related to gene-editing technology and the pioneering pig-to-human transplants currently underway in the USA and China, mean that xenotransplantation (XTx) may become a realistic option for patients waiting for human organ transplants in the foreseeable future. XTx policy needs to be informed by understanding what the public thinks. We undertook what we believe to be the first representative population survey in the United Kingdom of attitudes and views towards XTx. Sixty-five percent of respondents were supportive. Fifty-nine percent would be willing to accept a transplant from a pig. The main concerns related to genetic modification and animal welfare. Public support was subject to rigorous oversight of safety and animal welfare by an independent regulator. There were significant differences in view related to age, sex, ethnicity, educational qualifications and religion with women, people from Black and Asian minority groups and Muslims less supportive of XTx. These findings highlight the importance of demonstrating to the public that XTx can be provided safely while ensuring high standards of animal welfare. We propose reproducing this survey in other European countries would enable comparative analysis and adapting it to incorporate professional opinion may help inform the development of XTx policy and practice in Europe.

## Graphical Abstract

**Figure:**
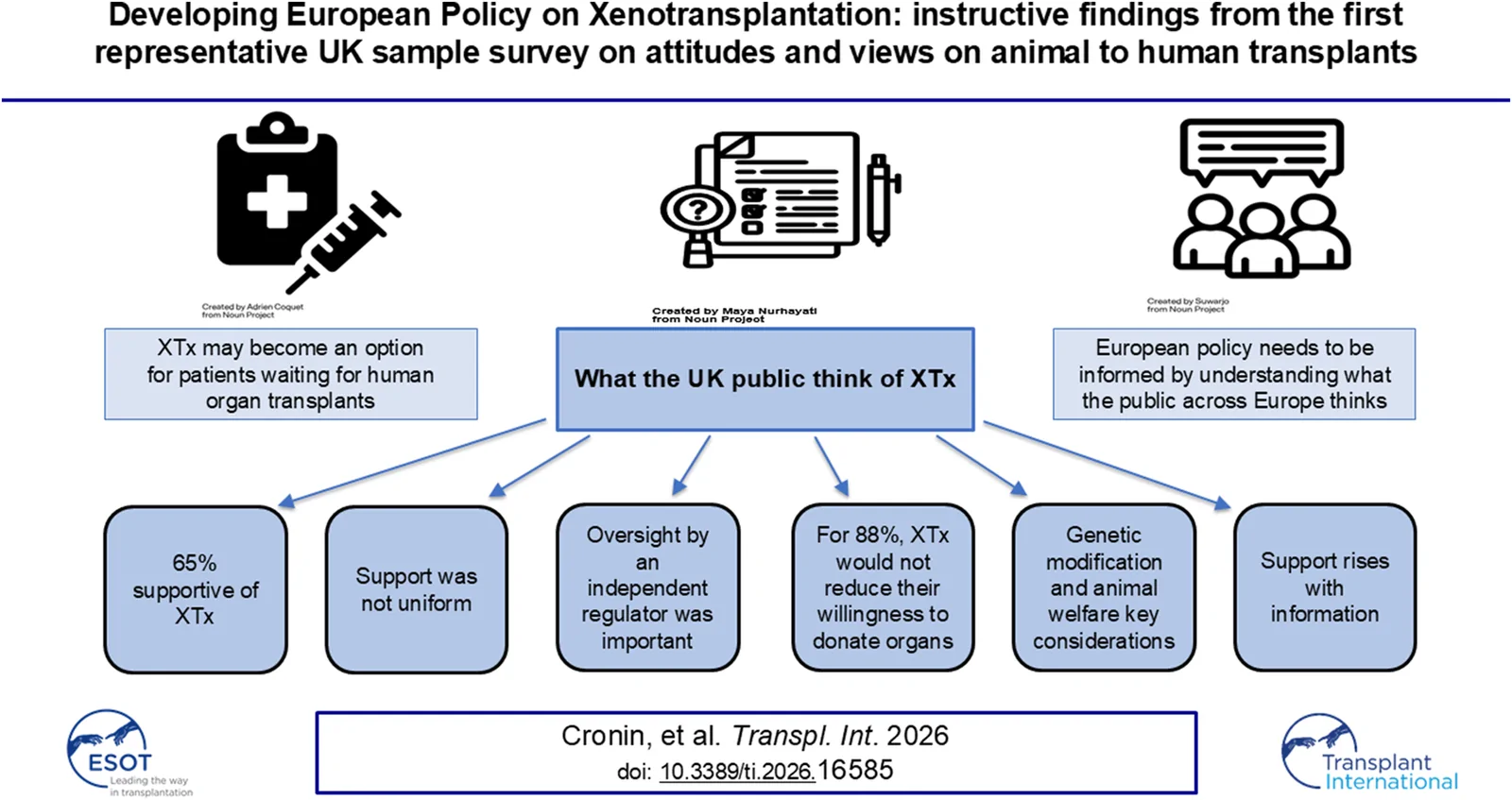

## Introduction

Pioneering European researchers have made a significant contribution to the field of XTx, which is the transplantation of living cells, tissues or organs from one species to another, most notably from pigs or non-human primates to humans [1].

In his article setting out in detail the journey of XTx from its infancy, Wayne Hawthorne describes the first published attempt of xeno-transfusion in Europe [2]. It took place in Paris, France, in the 17th century, when Jean-Baptiste Denis, a French physician, doctor of King Louis XIV, and Paul Emmerez, surgeon, transfused what has been assumed to be a small amount of blood from a lamb into a 15-year-old boy. According to records available, unfortunately, on the second attempted use of xeno-transfusion it proved unsuccessful and resulted in the death of the patient after which xeno-transfusion was outlawed by the French government [2, 3]. Subsequently, in 1906 the first reported successful kidney xenotransplant was carried out by Mathieu Jaboulay and Alexis Carrel from Lyon, France. They transplanted a pig kidney onto the brachial artery and cephalic vein of a 48-year-old woman. Immediately and reportedly for the first day and a half significant urine output was seen, but on the third day, they were forced to remove the kidney because of vascular thrombosis, almost certainly caused by what we now know to be hyperacute rejection [2]. Of course, at the time there was little understanding of immunology, never mind cross-species transplantation. But it is really over the last century that advances in medical science and specifically non-human primate studies and the advent of gene editing techniques that have brought the prospect of XTx closer to reality.

In 1969 the Nobel winning transplant immunologist Peter Medawar hypothesised that:

“*We should solve the problem of organ transplantation by using heterografts [xenografts] one day if we try hard enough and maybe in less than 15 years*” [4].

In 1984 surgeon Leonard Bailey performed the first cross-species infant heart transplant when he transplanted a baboon heart into a human infant known as ‘Baby Fae’. Despite ABO-incompatibility between patient and graft, the baboon heart functioned well for 2 weeks, but then underwent rejection, the baby sadly dying on day 20 [5]. In 1993, Tom Starzl and his team in Pittsburgh published a landmark case report in the Lancet of a baboon-to-human liver transplant [3]. In 1996, the Nuffield Council on Bioethics Report on XTx quoted a prediction from a UK biotechnology company that the first pig-to-human XTx would take place that year [6].

Since then, scientific research has focused on overcoming key outstanding immunological and infectious challenges related to XTx. Specifically, the immune mechanism underpinning hyperacute rejection and the infection risks associated with porcine endogenous retroviruses.

Alpha -1,3-galactosyltransferase or Alpha-Gal, is a carbohydrate found in most mammalian cell membranes, but importantly not in humans. It is the presence of Alpha-Gal in pigs that causes hyperacute rejection after transplantation into a human. Hyperacute meaning that the rejection occurs almost immediately in a matter of minutes to hours [7].

Porcine endogenous retroviruses or PERVs, are integrated into the genome of all pigs and cannot be eliminated like other viruses can and can infect human cells. And like other retroviruses (for instance HIV) they can induce tumours and immunodeficiencies. Current opinion is that PERVs are not likely to be pathogenic to humans, but the impact of cross-species infection remains to be elucidated and therefore represents a potential safety risk [7–9].

Until now Alpha-Gal and PERVs have posed major barriers to progress in XTx, Alpha-Gal because of hyperacute rejection and PERVs because of patient safety. However, the advent of gene editing technology has changed all that.

Gene editing technology and in particular the CRISPR Cas-9 gene editing tool enables scientists to edit parts of the genome with extraordinary precision by removing, adding or altering sections of the DNA sequence. In the CRISPR Cas9 system the Cas9 molecule, which is essentially a type of enzyme called an endonuclease, acts as a pair of molecular scissors that can cut the two strands of DNA at a specific location in the genome so that bits of DNA can be removed (knocked out) or added (knocked in). So, in recent years it has become possible to create gene edited pigs without Alpha-gal and with inactivated PERVs. This has been the key to XTx. In the 10-gene edited pig, 6 human genes are added or ‘knocked in’ to the pig genome to ensure it has the best chance of being accepted in a human recipient and four porcine genes are inactivated or “knocked out” to reduce the risk of infection, rejection and to moderate organ growth [10–15].

These advances in gene-editing technology and a handful of pioneering, genetically modified pig-to-human transplants in the United States (US) and studies in China suggest XTx is likely to become a realistic option for patients listed waiting for human organ transplants. Indeed, in 2025 the US Food and Drug Administration (FDA) approved the first clinical trial in XTx. Six people with end-stage kidney disease will receive kidneys from pigs that have been genetically modified [11]. The idea of enhanced survival with unparalleled equitable access to organs is tantalising and these technical feasibility studies are anticipated to provide invaluable data about highly relevant clinical outcomes, including structural, functional, immunological and infection outcomes. However, it is broadly agreed that there remain a good deal of unknown and indeed unknowable risks without an expanded programme of first in human studies, for instance the risk of zoonoses and the long-term impact of genetic modifications, and it is acknowledged that these technical advances also raise concerns about the implications for global health equity given the likely costs and other ethical and legal issues [4, 16, 17]. Moreover, for the enterprise of XTx to be realised as a global success it requires rigorous evaluation and understanding of XTx as a value-based proposition which takes account of decision-making driven by personal, ethical, social or cultural beliefs, in addition to technical feasibility studies. It requires understanding of the attitudes of patients and the broader public, and the level of risk that is acceptable to these groups.

Several surveys have been conducted internationally since the 1990s to establish the views on XTx of patients on the waiting list for organ transplants (see [18] for example), organ recipients (see [19] for example), healthcare staff (see [20] for example) and university students (see [21] for example). Now, with XTx approaching as a possible treatment option for patients with organ failure, it is important for policymakers and clinicians to understand how the public views this prospect. This is currently limited by the dearth of evidence from large-scale representative surveys. A recent meta-analysis of views concerning XTx showed the overwhelming majority of research published until very recently had been conducted on subgroups of the population, rather than the general public [22]. Since the publication of this meta-analysis, a 2023 survey of the public has been published in the US. [23]. To our knowledge, no similar research has been conducted elsewhere. To this end, we undertook what we believe to be the largest and most up-to-date survey in Europe of the United Kingdom (UK) public’s attitudes and views on XTx, focusing on the future transplantation of solid organs such as kidneys and hearts. The findings of the survey were intended to inform the UK Department of Health and Social Care’s Implementation Steering Group for Organ Utilisation (ISOU) Xenotransplantation Sub-Group (XTx Sub-group) set up in 2023 to provide independent advice to support the UK policy response to emerging XTx technology, but they may also be instructive for European policymakers [24]. In this paper, we expand on the findings of the survey that we originally presented in a brief research correspondence in the Lancet [25], as well as elaborate on their implications, as presented by AC at the 6th ELPAT Congress, held in Seville, Spain, in October, 2025.

## Materials and methods

### Survey development

The starting point for identifying relevant issues to explore was the seminal work on the ethics of XTx conducted in the 1990s by the Nuffield Council on Bioethics [6]. It invited submissions from a wide range of parties expected to have an interest in the topic while recognising that this did not represent the views of the public. The main issues explored were animal welfare-related concerns (both in relation to the principle of using either primate or non-primate animals for medical purposes, and the regulations that would need to be in place for such procedures to take place); the risk of transmission from animals to humans of infectious diseases as a consequence of these procedures; the criteria to select patients to be included in the early trials; and the possible effects on the healthcare system and recipients. We identified a recent meta-analysis of surveys of public views of XTx by Mitchell et al, [22] and looked at the questionnaires of the included studies. It was a useful starting point, but none of the questionnaires provided sufficient basis for our planned questionnaire development. Three of the researchers (AC, NM and MAH) were also observers on the XTx Sub-group, which allowed them to identify the issues and related questions raised in the Sub-group’s discussions. We also shared the content of the proposed survey with the XTx Sub-group and individual members for comment, whilst retaining our independence as researchers in deciding the topics and questions to include. Furthermore, we convened three lay advisory group meetings at intervals during the process to help develop the questionnaire, each with a group of five public contributors. These meetings helped us gauge general reactions to the prospect of XTx and identify contributors’ main concerns as they learned about XTx. This, in turn, informed which key questions to include in the survey. Public contributors also provided feedback on draft questionnaires, advising us on language, highlighting inconsistencies and pointing out areas lacking clarity.

We developed over 40 questions on attitudes to organ donation, religious and moral beliefs, attitudes to scientific and medical issues, prior knowledge of XTx and attitudes to transplantation of animal organs into humans (see Supplementary Appendix 1, pages 1-10, for the survey questionnaire).

As these were new questions, we commissioned the National Centre for Social Research (NatCen) to undertake cognitive testing of the questions. This consisted of 18 interviews conducted with a purposive sample of members of the public selected based on age, sex, educational level, their views on the use of animals for medical research and their dietary restrictions owing to religious or ethical beliefs. Interviewers probed interviewees’ comprehension of the terms used, rationale for the answers provided, ability to recall the information requested and comprehensiveness of their responses.

### Information provision as part of the survey completion

As we expected this topic to be new to most respondents completing the survey, we included information about the way in which XTx was likely to take place were it to be introduced. This was given in short paragraphs interspersed between survey sections that followed a first “uninformed” set of questions designed to ascertain respondents’ prior knowledge and unprompted views. After this initial set of questions, it was explained to respondents that organs would be sourced from genetically modified pigs, specially reared for organ donation and transplantation. A concise description of their living conditions and infection screening prior to donation was also provided. In our explanation of XTx, we ensured information provided reflected current scientific knowledge based on feedback from the XTx Sub-group. Additionally, guidance from the public contributors helped us maintain a neutral stance, neither advocating for nor against XTx. The wording was further tested for neutrality as well as ease and accuracy of interpretation as part of the cognitive testing undertaken by NatCen.

### Survey sample

We recruited the respondents from the Opinion Panel [26] of NatCen. Panel members in Great Britain were recruited from the British Social Attitudes (BSA) survey, while those living in Northern Ireland were recruited from the Life in Northern Ireland (LNI) survey. We opted for a random probability panel (where respondents are recruited through random sampling from the population) in line with evidence that they are more representative of the underlying population, thereby enabling more accurate estimates than non-probability (opt-in) panels [27]. Recruiting respondents from an existing online panel enabled us to use some of the information already available on members in our analysis, without adding to respondent burden. This comprised sociodemographic information, including sex (female or male), age group, highest educational qualification achieved, region of residence and self-described ethnicity. These data (apart from sex and ethnicity) are updated every 6 months.

Our sample size calculations indicated that a sample size of 3,000 would allow us to look at differences in responses between sub-groups with reasonable confidence such as between those who had “opted-in” to donating their organs on the organ donor register (41% of the adult population in the UK at the time) [28] and those who had not. This sample size gave us 80% power of detecting a 5% difference at the 95% confidence level.

A random sub-sample was selected from the Opinion Panel, maintaining the probability-based design. Odds of selection were adjusted based on extent to which a Panel member had characteristics that were over- or under-represented in the full Panel sample, improving the representativeness of the issued sample. The model used information on Panel members’ age, sex, region, household structure, income, education, economic activity, ethnicity, tenure, social class, interest in politics and party support, and their odds of selection were adjusted accordingly. Separate samples were taken from BSA and LNI, and each was stratified by the variables used in weighting for non-response. NatCen uses weighting to take account of non-response at all stages of running its Opinion Panel surveys from its initial recruitment to the Panel through attrition over time and to the current XTx survey (see Supplementary Appendix 2, page 11 for additional details on how weights were calculated).

### Data collection

The survey was administered by NatCen between 30 August and 29 September 2024. Panel members were initially invited to participate online, and sent reminders by post, email and/or text message. If they had not completed the survey after 2 weeks (and if telephone numbers were available), they were then contacted by phone to encourage online completion or offer an interview over the phone. A £5 high street voucher was sent to those who completed the survey as a “thank you”.

### Data analysis

We used Stata Standard Edition version 18 for the data analysis. Frequency distributions and weighted percentages were used to describe respondents’ characteristics, their attitudes and views towards XTx. Apart from the frequency distributions (numbers), all the percentages presented in this paper are weighted to help minimise bias due to differential non-response among sub-groups (Supplementary Appendix 2, page 11). We recoded the responses to the question “Do you believe that animal to human transplantation, as described before, should be allowed?” into two categories - i.e. supportive = (’Definitely should be allowed’ and ‘Probably should be allowed’) and unsupportive of XTx = (“Probably should not be allowed’ and ‘Definitely should not be allowed”). We cross-tabulated this new variable with demographic and other attitudinal variables, and Person’s Chi squared statistics and p-values were estimated to assess the strength of the statistical association between the variables.

## Results

The overall response rate was 53% (3,193/6,024), though the numbers answering individual questions varied since respondents could choose not to answer questions. Supplementary Appendix 3 shows respondent characteristics. Compared to the general population in the 2021 Census [29], there was good representation by sex, age group, educational qualifications except for those with no qualifications (9% vs. 18%) and region. There was overrepresentation of people who self-described as ethnically White (89% vs. 82%) compared to Asian (7% vs. 9%) and Black (2% vs. 4%) people, overrepresentation of people with no religion (44% vs. 37%) and underrepresentation of Muslims (3% vs. 7%). Non-response bias is unlikely to be a limitation since the demographic characteristics of panel members invited to the research closely align with those who completed the survey (see Supplementary Appendix 3, pages 12–13).

Sixty-five percent of respondents stated that they were aware of the possibility of XTx. The predominant ‘gut reaction’ to the possibility, before receiving any information, was a mix of uncertainty, concern and nervousness (see Figure 1).

**FIGURE 1:**
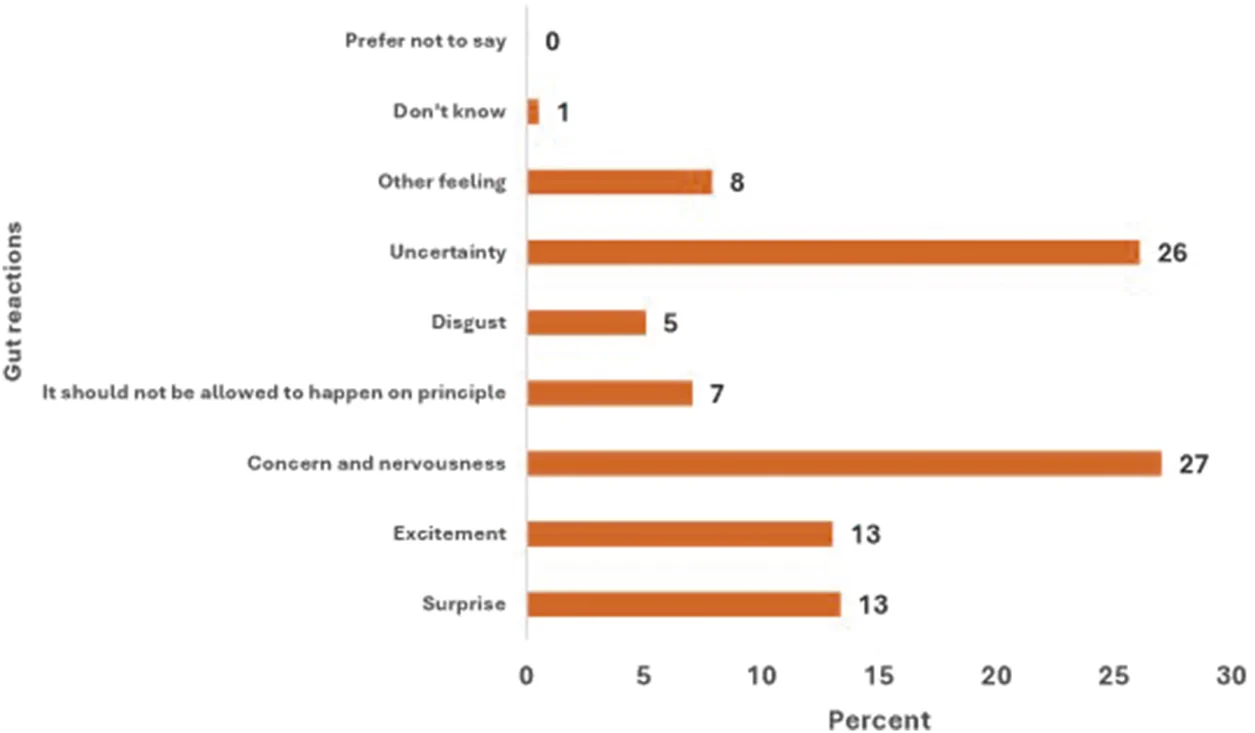
What is your gut reactions to the idea of animal to human organ transplants? (n=3,193), multiple responses allowed.

However, the majority (65%) were supportive (i.e. 19% responding that it should definitely be allowed and 46% that it should probably be allowed). Twenty-four percent stated that XTx should probably not be allowed and 11% that it definitely should not be.

Support for XTx was significantly (p < 0.001) higher among men (74%) than women (57%); among those self-identifying as White (67%) than the Black/Black British ethnic group (34%); and among those aged 60+ (69%) and in the 18–39 years age group (65%) than among those in the 40–59 years age group (62%). Also, support was significantly (p < 0.001) lower among Muslims (42%) than those with no religion (68%), Christians (65%) and other religious groups (54%); among vegans (26%), and among people with a disability that affects their day-to-day life a lot (57%) (see Supplementary Appendix 4, pages 14–15). Furthermore, support for XTx was significantly (p < 0.001) higher among those who had thought about deceased organ donation and had recorded their wish to donate on the National Health Service (NHS) Organ Donor Register (73%) compared to those who had thought about deceased organ donation but could not remember the decision they had entered in the NHS Organ Donor Register (69%), or opted out (54%), those who have not registered a decision as yet (62%) and those who have not thought about deceased organ donation (52%). Those who were aware of the possibility of transplanting animal organs into humans before the survey were more likely to support XTx (75%) than those who were not aware before the survey (47%). Support was significantly (p < 0.001) higher among those who were willing to accept an organ transplant from a pig if needed (91%). However, approximately 1 in 4 (28%) respondents not willing to accept an organ from a pig were nevertheless willing for XTx to be made available. Also, support for XTx was higher among those who indicated that availability of XTx would not affect their willingness to donate their organ(s) after death (69%) than those who indicated that the availability of XTx would increase (43%) or decrease (37%) their willingness to donate their organ(s) at the end-of-life. There were no significant differences in support for XTx by region and in terms of whether a person had had experience of organ donation either personally, within the family or via a close friend (see Supplementary Appendix 4, pages 14–15).

The level of support for XTx was higher at the end of the questionnaire after basic information on the likely implementation of XTx had been provided rising from 65% to 67% with the proportion unsupportive falling by about 6% (see Figure 2). The increase was statistically significant at the 1% level (p < 0.001). The provision of information resulted in a change in the composition of those who were undecided, that is, neither supporting nor opposing XTx. This group accounted for about 5% of respondents, comprising 7% of those who had previously been unsupportive and 3% of those who had originally been in the supportive sub-group.

**FIGURE 2:**
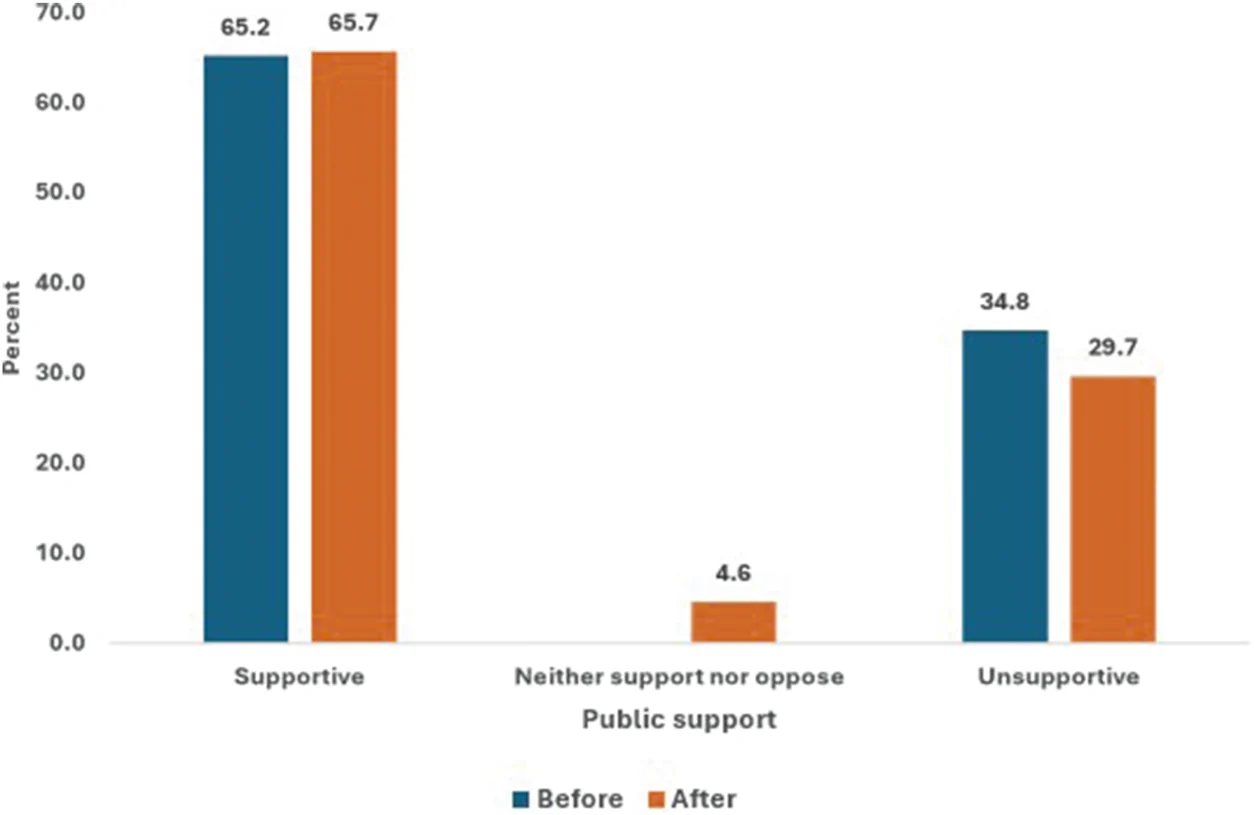
Which of the following best describe your support in principle for animal to human organ transplants (after information provided)? (n=3,173). Note: Analysis was limited to respondents who answered both before and after questions. Chi-squared statistic=2700, p<0.001.

Among those who were supportive of XTx, the main reason given was to save lives that would otherwise have been lost (34%) (see Figure 3). Among those who thought that XTx should not be allowed, the main reasons given were that animals should not be genetically modified (31%) or kept in sterile conditions indoors (24%), solely to provide organs for human transplantation (see Figure 4).

**FIGURE 3:**
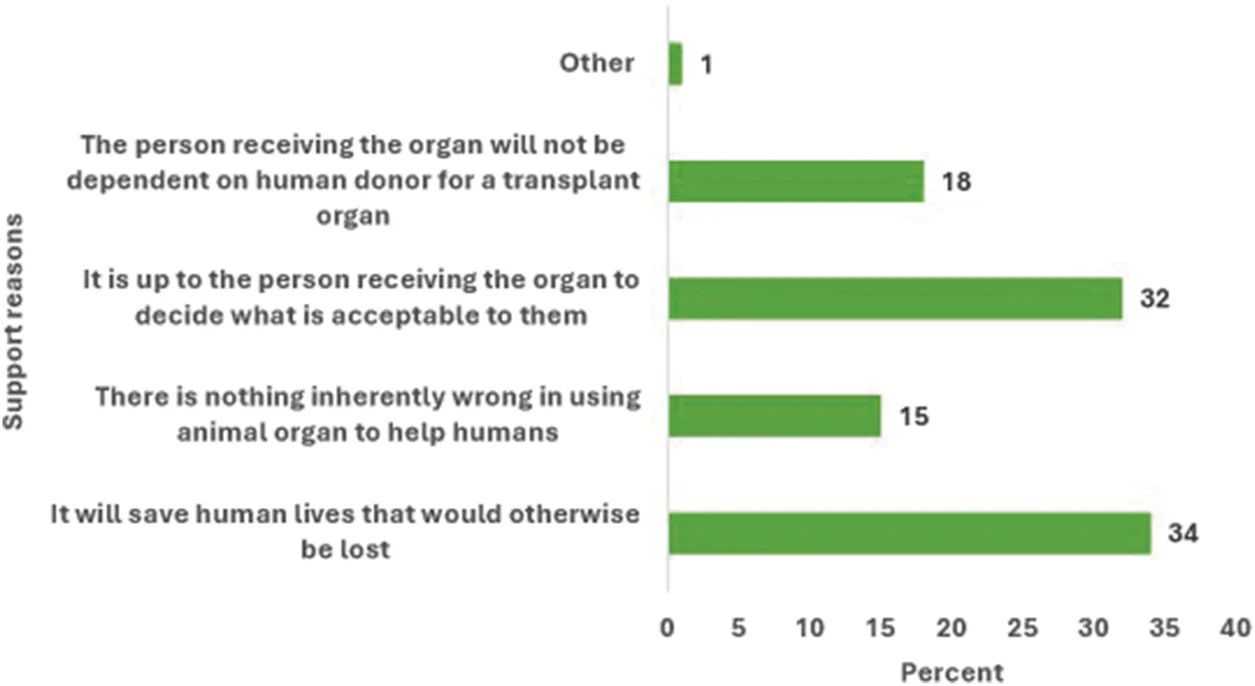
Why do you think animal to human transplantation should be allowed? (n=2,103), multiple responses allowed. Note: This analysis is limited to only those who were supportive of XT. Reported percentages are the percentage of responses that sum up to 100%.

**FIGURE 4:**
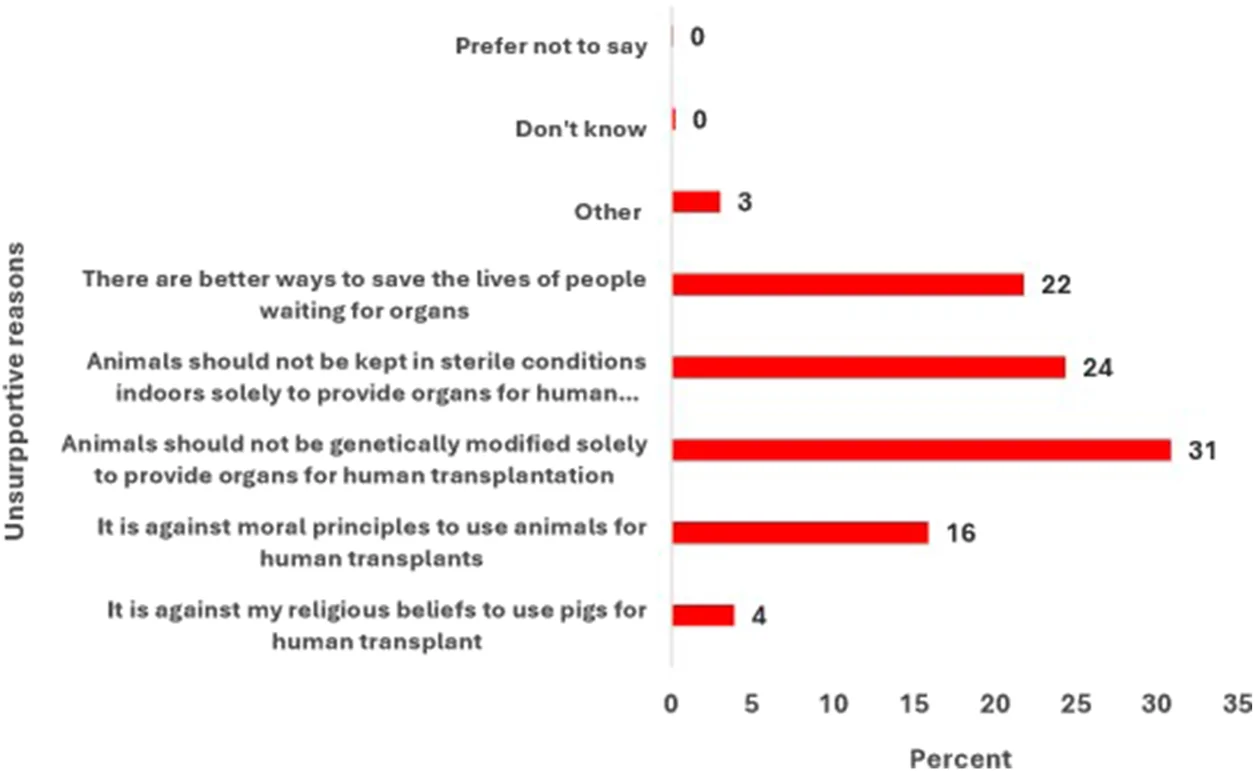
Why do you think animal to human transplantation should NOT be allowed? (n=1,070), multiple responses allowed. Note: This analysis is limited to those who were unsupportive of XT. Reported percentages are the percentage of responses that sum up to 100%.

Fifty-nine percent of the sample would be willing to accept a transplant from a pig, but this proportion was significantly (p < 0.001) lower among those who did not eat meat (36%) or who did not eat pork (24%). Muslims were significantly (p < 0.001) less likely than other religious groups to be willing to accept a pig transplant even if their chances of survival before a human organ became available were low (17%).

The main reason given for accepting a pig organ was if the chances of survival before a human organ becomes available were low (see Figure 5). The main concerns were related to mixing animal and human DNA (32%) and risks of infection from animals (24%), though a similar proportion (32%) reported none of these concerns (see Figure 6).

**FIGURE 5:**
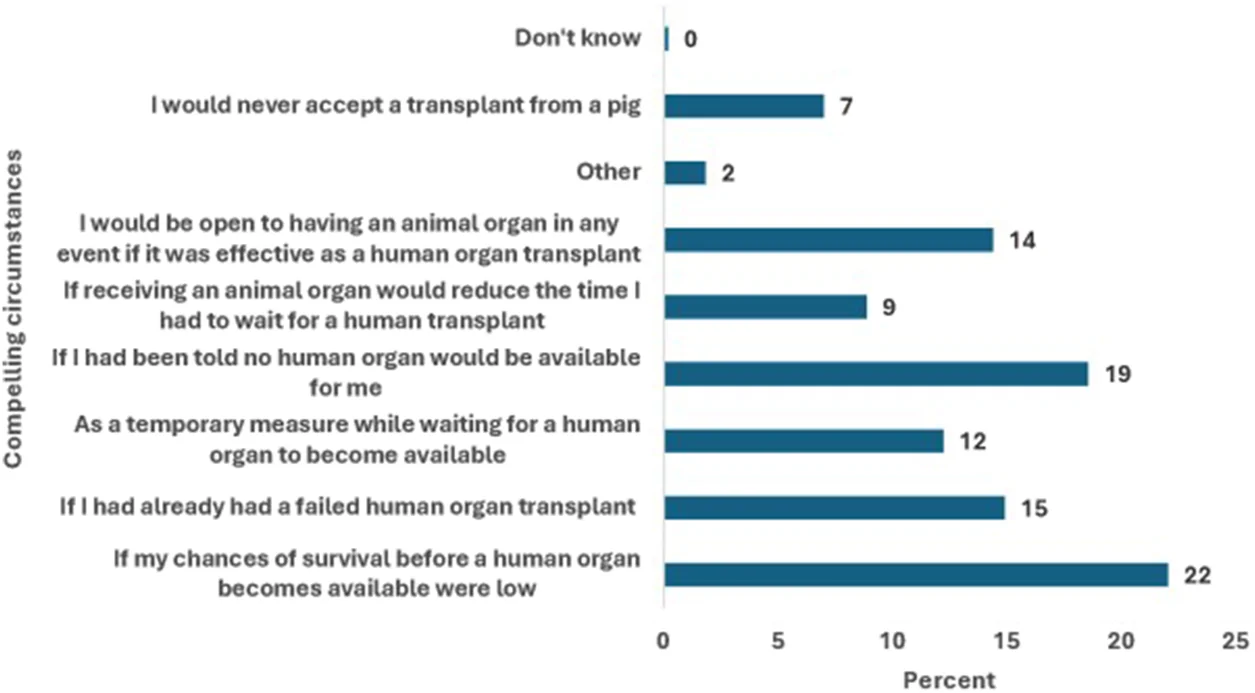
In which circumstance, if any, would you be willing to accept a transplant from a pig? (n=3,193), multiple responses allowed. Note: Reported percentages are the percentage of responses that sum up to 100%.

**FIGURE 6:**
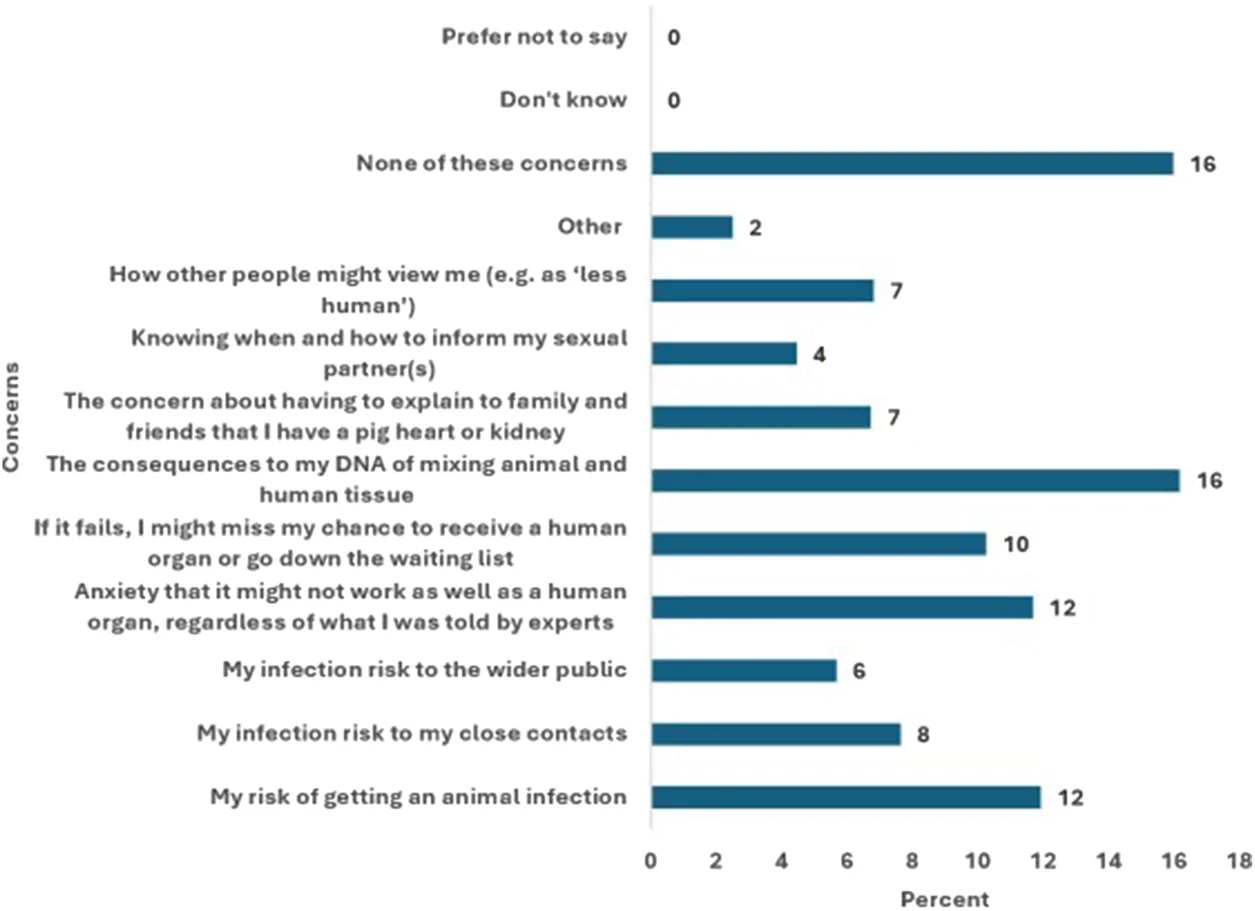
Which of the following concerns would you have if you were offered a transplant from a pig? (n=3,193).Note: Reported percentages are the percentage of responses that sum up to 100%.

Approval of the specific organ and procedure by an independent organisation similar to the Medicines and Healthcare product Regulatory Agency (MHRA), was the measure most respondents felt would encourage them to consider accepting an animal organ (see Figure 7).

**FIGURE 7:**
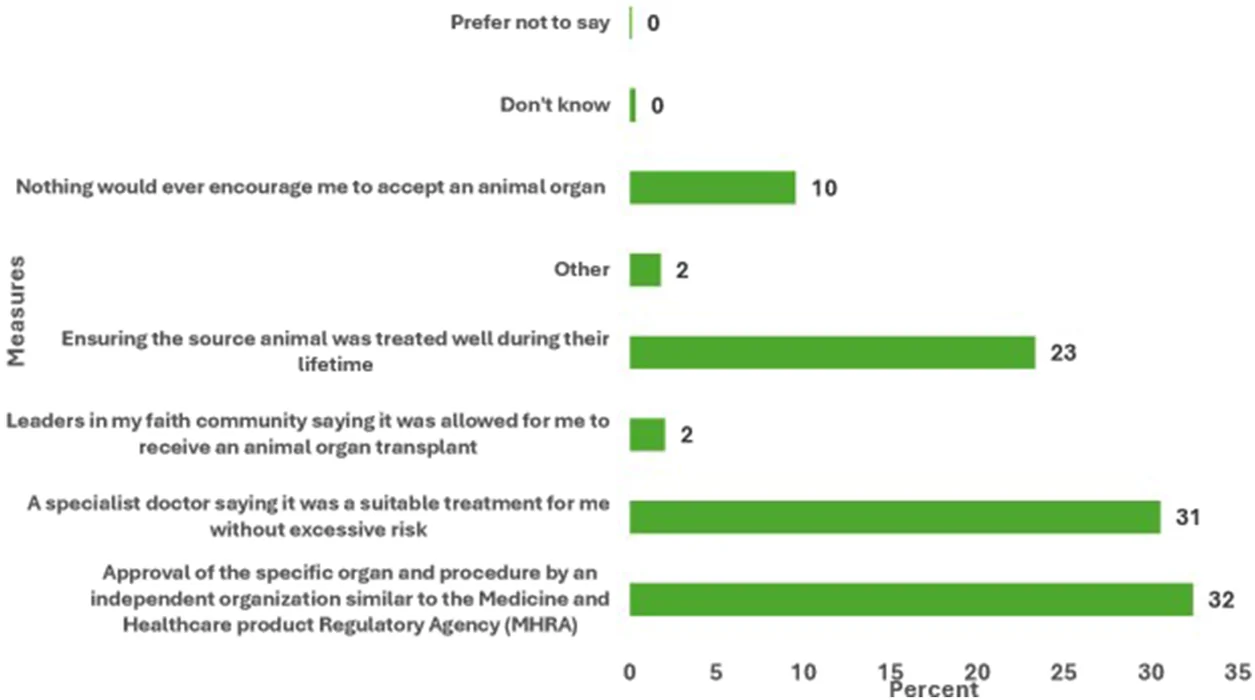
If you needed a transplant, what measures would encourage you to consider opting for an animal organ? (n=3,193), multiple responses allowed. Note: Reported Percentages are the percentage of responses that sum up to 100%.

The public’s threshold regarding when XTx should be made available on the NHS was high, with 41% indicating XTx should only be introduced once it had become as successful as human organ transplantation. Respondents indicated that patients who had waited the longest for a suitable human organ should be a high priority for animal-to-human organ transplants (if proven to be safe and effective). Patients who had already received an animal organ, however, were seen by most respondents (58%) as a lower priority for a human organ transplant provided their animal-to-human organ transplant was functioning satisfactorily.

Lastly, the prospect of a new supply of organs for transplants did not seem to affect views on current organ donation at the end-of-life, with 88% of respondents indicating that it would not affect their willingness to donate their organs and only 5% reporting that it would reduce their willingness.

## Discussion

Our survey analysis indicates majority UK public support for XTx which increased with the provision of basic information on the likely way in which animal organs would be made available. However, public support is likely to be dependent on rigorous oversight by an independent regulator in relation to ensuring animal welfare, minimising the risk of zoonosis and assuring high clinical standards of care. This is broadly in line with the conclusions of two major UK reports published in the late 1990s when the prospect of XTx had previously been a focus of policy attention [6, 30]. However, though consulting widely, neither report drew on any public survey data. The current survey shows that beyond the overall majority support for XTx lie significant differences of view related to age, sex, ethnicity, educational qualification and religion which merit further exploration.

The UK survey findings had interesting similarities and differences compared with the representative population survey undertaken in the US in 2023. Forty-one percent of the UK sample stated that they would not accept a pig organ transplant. The US proportion was almost identical. Approximately 40% expressed discomfort with the prospect of receiving a pig organ, either for themselves or a loved one. As with the UK respondents, the majority indicated they would choose a pig organ transplant over waiting for a human organ for transplantation to become available, provided the pig organ transplant was equally effective. However, most US participants expressed increased comfort with the idea of accepting a xenotransplant when informed that the pig had been genetically modified [23] —an attitude that contrasts with the perspectives of UK respondents, who were less reassured by genetic modification but reassured by the oversight of an independent regulator. Consistent with the UK findings, characteristics such as being female and/or part of an ethnic minority group were also frequently associated with greater hesitation towards XTx in the US.

The strengths of the current study are that it is based on the most comprehensive and up-to-date survey of public attitudes toward XTx in Europe; it includes a large broadly representative sample of the UK population derived from a high quality probability-based survey panel; the questions cover issues identified both by experts and members of the public; the questions were subject to rigorous cognitive testing; the data were collected by NatCen, one of the UK’s leading social survey research agencies; and the analysis allows for a comparison of informed and uninformed public views. The main limitation is that the achieved sample under-represented those with no educational qualifications, members of ethnic minority groups and Muslims. In addition, a sample survey of this size cannot provide statistically meaningful estimates of the views of the smaller ethnic and religious minority groups in the UK population such as Jews and Buddhists. The possibility of adding a boost sample to capture the views of such groups was discussed at length but rejected on the grounds that results would not have been comparable to those derived from the main sample.

In policy terms, the survey findings indicate a need for transparency by providing the public with reliable information on all aspects of the XTx process over the next few years, assuming clinical translation progresses. This needs to be provided by independent researchers, and veterinary and clinical regulators (e.g. on the safety of the use of genetically modified pigs, the risk of infection from the pigs, the conditions in which the pigs are reared, the results of clinical trials, ethical issues arising, etc.). The finding that support for human organ donation did not appear likely to decrease if animal organs became available for transplantation potentially removes one major policy concern about the advent of XTx. However, the survey shows that there are sub-groups in the UK population who are sceptical and/or unconvinced about proceeding with XTx. Perhaps most importantly, the least supportive sub-groups appear to be among those with the highest level of unmet need for human organ transplants, such as self-identified Black and Asian people [31]. In part, this appears to be due to both groups having poorer experiences of healthcare and lower levels of trust in doctors and the government than other members of the public [32]. Further, in-depth interview-based research is needed to understand better the basis of these reservations. A further important issue not reported here relates to the criteria that the public would regard as acceptable and legitimate to determine which patients should have priority, initially for clinical trials and eventually for more routine healthcare (by, for example, the NHS in the UK) provision of XTx given that, even with access to pig organs, demand is likely to outstrip supply, not least given the cost of providing pig organs.

## Conclusion

The science and technology of XTxis evolving at pace. Recent landmark scientific and clinical advances inXTx mean that it may become a realistic option for patients listed waiting for human organ transplants in Europe in the foreseeable future. Policy in this area needs to be informed by understanding what the public thinks about this development. Using the findings from our survey, the first representative UK sample survey on public attitudes and views on animal to human transplants, may help to inform and develop a framework to shape xenotransplant policies in Europe. For example, reproducing this survey in other European countries would enable comparative analysis and adapting it to incorporate professional opinion would provide additional valuable insight into what are key challenges with future clinical translation.

Despite some nervousness at the prospect, findings from our survey suggest the majority of the UK public is supportive and this proportion rose after information was provided about how XTx is likely to be provided (i.e. using gene-edited pigs). Fifty-nine percent overall would be willing to accept a transplant from a pig. The main concerns among those who were not supportive related to genetic modification and animal welfare. It is clear that rigorous oversight of safety and animal welfare by an independent regulator is a prerequisite of public support for XTx in practice. In Europe, the European Medicines Agency is well placed to take on that role that will be fundamental in facilitating clinical trials in XTx, in which working group and stakeholder engagement will be key. The significant differences of view related to age, sex, ethnicity, educational qualification and religion identified in our UK representative sample deserve further investigation since women, people from Black and Asian minority groups and Muslims are less supportive of XTx in principle. These findings highlight the policy importance of demonstrating to the public that XTx can be provided safely while ensuring high standards of animal welfare. European policymakers must provide reliable public information on XTx and consider carefully how it should be delivered across Europe as the technology unfolds. Reproducing this survey in other European countries with representative samples to enable comparative analysis and adapting this survey to incorporate professional opinion across Europe is likely to be highly informative and may contribute to future development of a novel framework for XT thereby shaping future European policy and practice.

## Data Availability

The original contributions presented in the study are included in the article/Supplementary Material, further inquiries can be directed to the corresponding authors.
