# Supplementary material for: Developing European policy on xenotransplantation: instructive findings from the first representative UK sample survey on attitudes and views on animal to human transplants
Source: Transpl Int. 2026 Sep 15;39:16585. doi: 10.3389/ti.2026.16585 (PMC13619536; doi:10.3389/ti.2026.16585)
Supplement: Supplementary file 1 [file DataSheet1.pdf]

Appendices

Appendix 1: Survey questionnaire

MODULE 1: Generalised attitudes to organ donation

{ASK ALL}
XTIntro1

Now we will ask you some questions on human organ donation and transplantation.

{ASK ALL}
HumanODT
To what extent do you support or oppose **human organ donation and transplantation**?

INTERVIEWER: READ OUT

1. Strongly support
2. Support
3. Neither support nor oppose
4. Oppose
5. Strongly oppose

{ASK ALL}
ODonate
Have you thought about donating any of your organs for transplantation at the end of your life?

1. Yes
2. No

{ASK IF ODonate = 1}
ODRWish1
Have you registered your decision in relation to donating your organs for transplantation, at the end of your life, on the NHS Organ Donor Register?

INTERVIEWER: READ OUT

1. Yes
2. No
3. Can't remember

{ASK IF ODRWish1 = 1}
ODRWish2
What was the decision you registered on the NHS Organ Donor Register?

INTERVIEWER: READ OUT

1. I registered my willingness (opt-in) to donate some or all of my organs at the end of my life
2. I registered my unwillingness (opt-out) to donate any of my organs at the end of my life
3. I can't remember the decision I registered

{ASK ALL}
ODTExp
Do you, or a family member or close friend, have any experiences of organ donation or transplantation?

1. Yes
2. No

MODULE 2: Religious and moral beliefs

{ASK ALL}
XTRel
What is your religion?

INTERVIEWER: READ OUT

1. No religion
2. Christian (including Church of England, Catholic, Protestant and all other Christian denominations)
3. Buddhist
4. Hindu
5. Jewish
6. Muslim
7. Sikh
8. Any other religion (please describe)
9. Prefer not to say

{ASK ALL}
RelNFood
Do you consider yourself to be any of the following?

INTERVIEWER: READ OUT

1. Vegetarian {HL HELPLINK: a person who does not eat meat or fish}
2. Pescatarian [HL HELPLINK: a person who does not eat meat but does eat fish}
3. Vegan {HL HELPLINK: a person who does not eat or use animal products}
4. Mainly vegetarian but occasionally eat meat
5. Someone who eats meat, excluding pork for religious reasons
6. Someone who eats meat
7. None of these

MODULE 3: Attitudes to related scientific and medical issues

{ASK ALL}
GMMeat
To what extent do you support or oppose **genetically modifying animals to produce meat for human consumption**?

It does not matter if you consume genetically modified meat or not, we just want to know how you feel about it in principle.

INTERVIEWER: READ OUT

1. Strongly support
2. Support
3. Neither support nor oppose
4. Oppose
5. Strongly oppose

{ASK ALL}

AnimalUse

To what extent do you support or oppose **the use of animals in medical research for human benefit**?

INTERVIEWER: READ OUT

1. Strongly support
2. Support
3. Neither support nor oppose
4. Oppose
5. Strongly oppose

{ASK ALL}

AnimalMaterial

Human health care has used material derived from animals for many years, including insulin for diabetics, vaccines, and the use of pig heart valves in open heart surgery. To what extent do you support or oppose **the established use of animal tissue in NHS health care**?

INTERVIEWER: READ OUT

1. Strongly support
2. Support
3. Neither support nor oppose
4. Oppose
5. Strongly oppose

MODULE 4: **Prior knowledge of animal-to-human organ transplantation**

XTIntro

This questionnaire seeks your views on the transplant of organs (namely the heart and the kidneys) from animals to humans. The use of animal organs could potentially address the shortfall of human organs needed for transplant. However, it may have associated risks. Currently, pig organs are the most likely to be used in animal to human organ transplants.

As this is a new topic, we do not expect you to have prior knowledge. {IF WEB "We will shortly be presenting you with more information – please answer the following questions without checking or referring to any online sources.}

{ASK ALL}

XTAware1

Were you aware of the possibility of transplanting animal organs into humans before starting this questionnaire?

1. Yes
2. No

{ASK ALL}

XTReact [MULTICODE]

What is your gut reaction to the idea of animal to human organ transplants?

WEB: "Please select all that apply"
TEL: "INTERVIEWER: READ OUT EACH OPTION AND CODE ALL THAT APPLY"

1. Surprise
2. Excitement
3. Concern and nervousness
4. It should not be allowed to happen on principle
5. Disgust
6. Uncertainty
7. Other feeling (please describe).

{ASK ALL}

XTReact2

Please tell us why you feel this way.

OPEN

MODULE 5: **Attitudes to the transplantation of animal organs into humans**

{ASK ALL}

XAttitudesIntro

Genetically modified pigs would be specially reared for the purpose of organ donation for transplantation. These pigs would live indoors, in sterile conditions but in social groups. The organs removed from these modified pigs would be screened for known infections before being used for transplant.

{ASK ALL}

XTAllow

Irrespective of whether you would take it up yourself…

Do you believe that animal to human transplantation, as described before, should be allowed?

INTERVIEWER: READ OUT

1. Definitely should be allowed
2. Probably should be allowed
3. Probably should not be allowed
4. Definitely should not be allowed

{ASK IF XTAllow = 1 or 2}

XTAllowReas1 [MULTICODE]

Why do you think animal to human transplantation **should** be allowed?

WEB: “Please select all that apply”
TEL: “INTERVIEWER: READ OUT EACH OPTION AND CODE ALL THAT APPLY”

1. It will save human lives that would otherwise be lost
2. There is nothing inherently wrong in using animals to help humans
3. It is up to the person receiving the organ to decide what is acceptable to them
4. The person receiving the organ will not be dependent on human donors for a transplant organ
5. Other (please describe)

{ASK IF XTAllow = 3 or 4}

XTAllowReas2 [MULTICODE]

Why do you think animal to human transplantation **should not** be allowed?

WEB: “Please select all that apply”
TEL: “INTERVIEWER: READ OUT EACH OPTION AND CODE ALL THAT APPLY”

1. It is against my religious beliefs to use pigs for human transplants
2. It is against my moral principles to use animals for human transplants
3. Animals should not be genetically modified solely to provide organs for human transplantation
4. Animals should not be kept in sterile conditions indoors solely to provide organs for human transplantation
5. There are better ways to save the lives of people waiting for organs
6. Other (please describe)

MODULE 6: **Personal acceptability of xenotransplantation and views on risks**

{ASK ALL}

XAcceptIntro

Kidney and heart transplants using human organs can save lives. However, there is a shortage of suitable human organs for transplants. This scarcity is especially acute for children. Over 7,000 people in the UK are waiting for a transplant, with three people dying each day while waiting.

Although doctors are still some way away from being able to successfully transplant animal organsinto humans, one of the possible risks is the transfer of known or unknown infections. It

is also conceivable that close contacts of the patients who have received an animal organ transplant could become infected. This means that it may be necessary to monitor patients and their close contacts at intervals after the transplant.

{ASK ALL}

XTAcceptA

If you needed an organ transplant, would you be willing to accept one from a pig?

1. Yes
2. No

{ASK ALL}

XTAcceptB [MULTICODE]

In which circumstances, if any, would you be willing to accept a transplant from a pig?

WEB: “Please select all that apply”
TEL: “INTERVIEWER: READ OUT EACH OPTION AND CODE ALL THAT APPLY”

1. If my chances of surviving before a human organ became available were low
2. If I had already had a failed human organ transplant
3. As a temporary measure while waiting for a human organ to become available
4. If I had been told no human organ would be suitable for me
5. If receiving an animal organ would reduce the time I have to wait for a human organ transplant
6. I would be open to having an animal organ in any event as long as it was as effective as a human organ transplant
7. Other (please describe)
8. I would never accept a transplant from a pig [EXCLUSIVE]

{ASK ALL}

XTRecieveA [MULTICODE]

Which of the following concerns would you have if you were offered a transplant from a pig?

WEB: “Please select all that apply”
TEL: “INTERVIEWER: READ OUT EACH OPTION AND CODE ALL THAT APPLY”

1. My risk of infection from the animal
2. My infection risk to my close contacts
3. My infection risk to the wider public
4. Anxiety that it might not work as well as a human organ, regardless of what I was told by experts
5. If it failed, I might miss my chance to receive a human organ or go down the waiting list
6. None of these concerns [EXCLUSIVE]

{ASK ALL}

XTRecieveB [MULTICODE]

What other personal concerns would you have, if any, if you were offered a transplant from a pig?

WEB: “Please select all that apply”
TEL: “INTERVIEWER: READ OUT EACH OPTION AND CODE ALL THAT APPLY”

1. The consequences to my DNA of mixing animal and human tissue
2. The concern about having to explain to family and friends that I have a pig heart or kidney
3. Knowing when and how to inform any sexual partner(s)
4. How other people might view me (e.g. as 'less human')
5. Other (please describe)
6. None of these concerns [EXCLUSIVE]

{ASK ALL}

XTMeasures [MULTICODE]

If you needed a transplant, what measures would encourage you to consider opting for an animal organ?

WEB: "Please select all that apply"
TEL: "INTERVIEWER: READ OUT EACH OPTION AND CODE ALL THAT APPLY"

1. Approval of the specific organ and procedure by an independent organisation similar to the Medicines and Healthcare products Regulatory Agency (MHRA)
2. A specialist doctor saying it was a suitable treatment for me without excessive risk
3. Leaders in my faith community saying it was allowed for me to receive an animal transplant
4. Ensuring the source animal was treated well during their lifetime
5. Other (please describe)
6. Nothing would ever encourage me to accept an animal organ [EXCLUSIVE]

{ASK ALL}

XTFollowUp

If you received an animal organ transplant, do you think your close contacts would be willing to be followed up by medical professionals over a long period of time?

INTERVIEWER: READ OUT

1. Most of my close contacts would agree to this
2. Some of my close contacts would agree to this
3. Most of my close contacts would not agree to this
4. None of my close contacts would ever agree to this

MODULE 7: **Treatment priorities, cost and efficacy**

{ASK ALL}

XTWaitList [MULTICODE]

Who should be invited from the waiting list to take part in early clinical trials designed to find out whether it would be safe and effective to use pig hearts or kidneys for transplantation?

WEB: "Please select all that apply"
TEL: "INTERVIEWER: READ OUT EACH OPTION AND CODE ALL THAT APPLY"

1. Adults

2. Children
3. Healthiest
4. Those who have waited the longest for a suitable organ
5. Those closest to death
6. Those whose previously transplanted human organ has now failed
7. Those who are unsuitable for a human organ
8. Anyone who volunteers

{ASK ALL}

XTPriority2 [MULTICODE]

If proven safe and effective, which groups, if any should be a high priority to be offered animal organ transplants?

WEB: “Please select all that apply”
TEL: “INTERVIEWER: READ OUT EACH OPTION AND CODE ALL THAT APPLY”

1. Adults
2. Children
3. Healthiest
4. Those who have waited the longest for a suitable organ
5. Those closest to death
6. Those whose previously transplanted human organ has now failed
7. Those who are unsuitable for a human organ
8. Anyone who volunteers

{ASK ALL}

XTPriorityRel

Some people may have a religious or moral objection to animal organ transplants. If animal to human organ transplants become available on the NHS, do you think these people should be given priority for human organs?

1. Yes
2. No

{ASK ALL}

XTAttitudeB

When animal organ transplants first become available, it is likely that they will be more expensive than human organ transplants and are unlikely to work as well initially. Over time it is hoped that the costs will drop, and the treatment will become as successful as human organ transplants.
Given this, when should animal organ transplants be made available on the NHS?

INTERVIEWER: READ OUT

1. As soon as they become available
2. Only after they become less expensive

3. Only after they become as successful as human organ transplants
4. Only after they become less expensive and as successful as human organ transplants
5. This treatment should not be made available on the NHS at all

{ASK ALL}
XTFuture
Given that for the foreseeable future, there will still be insufficient human and animal organs for transplantation, should a patient who has already received an animal organ transplant...

INTERVIEWER: READ OUT

1. Be a lower priority for a human organ while their animal organ is functioning satisfactorily?
2. Be an equal priority for a human organ while their animal organ is functioning satisfactorily?

MODULE 8: **Organ donation after death**

{ASK ALL}
XTODD1
How would the availability of pig organ transplants on the NHS affect your willingness to donate your organs after your death?

INTERVIEWER: READ OUT

1. It would **reduce** my willingness to donate my organs
2. It would **increase** my willingness to donate my organs
3. It would **not affect** my willingness to donate my organs

{ASK ALL}
XTODD2
How would the availability of pig organ transplants on the NHS affect your attitude to one of your loved ones donating their organs after their death?

INTERVIEWER: READ OUT

1. It would **reduce** my acceptance for them to donate their organs
2. It would **increase** my acceptance for them to donate their organs
3. It would **not affect** my acceptance for them to donate their organs

MODULE 9: **Priority of xenotransplantation over other approaches**

{ASK ALL}
XTPriority [MULTICODE]

Given that genetically modified pig organs will be expensive should any of the following options receive more funding from the NHS ahead of animal to human organ transplantation?

WEB: “Please select all that apply”
TEL: “INTERVIEWER: READ OUT EACH OPTION AND CODE ALL THAT APPLY”

1. Preventing heart and kidney disease in the first place
2. Increasing the number of human organs available from deceased donors
3. Improving the use of the human organs which are found to be unusable
4. None of the above [EXCLUSIVE]

MODULE 10: **Follow-up research on xenotransplantation**

{ASK ALL}
XTFollowUp
Have your views on the use of animal organs for transplant in humans changed since you started completing this survey?

1. Yes
2. No

{ASK IF XTFollowUp = 1}
XTFollowUpA
Which of the following statements best describes your views now on the use of animal organs for transplant in humans?

It does not matter if you would be willing to receive an animal organ transplant or not, we just want to know your views on animal to human organ transplant in principle.

INTERVIEWER: READ OUT

1. Strongly support
2. Support
3. Neither support nor oppose
4. Oppose
5. Strongly oppose

{ASK ALL}
XTFollowUpFuture
Can we contact you at some point in the future to invite you to take part in further research to understand your views on this topic in greater depth?

1. Yes
2. No

Appendix 2: NatCen's approach to weighting survey responses

Non-response to NatCen Opinion Panel surveys can occur at various points: the recruitment survey, the invitation to join the panel (at the end of the recruitment interview), subsequent attrition from the Panel, and the survey of panel members itself. The recruitment surveys are already weighted to adjust for non-response, and we compute further weights to adjust for non-response at the subsequent stages. The final weight is the product of these weights. This multi-stage approach is ideal because the correlates of non-response can be different at each stage.

These are the three weights computed:

1.Recruitment survey weight: The weights from the recruitment surveys followed similar designs: selection weights to adjust for uneven selection probabilities; non-response weights computed via logistic regression models of response (at address level) to adjust for differential non-response; calibration to population estimates.

2. Sampling weight: this weight adjusts for selection probabilities used in the sampling process and all non-response/attrition that occurs after the recruitment surveys but prior to sampling.

First, a logistic regression model was created to derive non-response weights to adjust for non-response that occurred prior to sampling i.e. at the panel recruitment stage plus any subsequent attrition. The following variables were used as predictors in the model: age and sex groups, region, household type, household income, education level, ethnicity, tenure, social class group, economic activity, political party identification, and interest in politics. The non-response weight was the inverse of the probability of joining/remaining in the panel.

As described above, a random subsample of panel members was selected for this survey. Weights were used to adjust the probabilities of selection, therefore a "sample selection" weight was computed to account for these differential selection probabilities (equal to the inverse of the probability of being selected for the sample). The final "sampling weight" is the product of the recruitment survey weight, the panel non-response weight and the sample selection weight.

3. Survey weight: this weight is used to adjust for non-response to this panel survey.

A logistic regression model was used to estimate the probability of response for each panellist issued to the survey. The panel survey weight was equal to the inverse of the probabilities of response. This weight adjusts for non-response using the same variables as used for the panel recruitment weight above i.e. age and sex groups, region, household type etc.

Two different models were used for the BSA respondents, one for BSA15-19 (the BSA face-to-face surveys) and one for BSA20-23 (the BSA push to web surveys). Another model was used for LNI respondents with predictors equivalent to those used in the BSA models. In each case, the resulting survey weight was multiplied by the sampling weight to create the final set of weights. The weights for LNI and BSA respondents were scaled before they were combined so that the proportion of respondents from NI and GB respectively is in line with the UK population.

Appendix 3: Characteristics of panel members invited to the survey and those who participated

Demographic characteristic	Issued (N)	Participated (N)	Issued (%)	Participated (%)
**Sex**				
Male	2814	1521	47%	48%
Female	3169	1671	53%	52%
Other	3	1	0%	0%
**Age**				
18-24	545	205	9%	6%
25-34	1041	431	17%	14%
35-44	975	497	16%	16%
45-54	1030	582	17%	18%
55-64	977	617	16%	19%
65+	1397	854	23%	27%
**Region**				
North East	255	145	4%	5%
North West	636	318	11%	10%
Yorkshire and Humberside	498	272	8%	9%
East Midlands	430	238	7%	7%
West Midlands	490	270	8%	8%
East of England	575	313	10%	10%
London	746	386	12%	12%
South East	787	434	13%	14%
South West	517	300	9%	9%
Wales	268	138	4%	4%
Scotland	484	254	8%	8%

Northern Ireland	300	125	5%	4%
**Highest qualification**				
Degree or equivalent, and above	2014	1082	34%	34%
A-levels/SCE Highers or vocational level 3 or equivalent, and above	1856	1002	31%	32%
Qualifications below A-levels or vocational level 3 or equivalent such as GCSE/O-Level/Standard Grade	1396	736	23%	23%
Other qualification	95	54	2%	2%
No qualifications	583	296	10%	9%
**Household structure**				
1 adult (no children)	925	529	17%	18%
1 adult (with children)	211	93	4%	3%
2 adults (no children)	2240	1266	41%	43%
2 adults (with children)	1037	502	19%	17%
3+ adults (no children)	1093	573	20%	19%
**Ethnicity**				
Any White background	5231	2835	88%	90%
Any Asian background	360	181	6%	6%
Any Black background	137	57	2%	2%
Mixed or other background	195	93	3%	3%
**Health condition/disability**				
Disability/health condition that affects daily life	1365	687	23%	22%
Disability/health condition that doesn't affect daily life	681	401	11%	13%
No disability/health condition	3918	2093	66%	66%

Appendix 4: Factors associated with support for XT

Factor	Category	Number	Supportive (%)	Unsupportive (%)	Chi-square value	P-value
**Sex*** **						
	Female	1,679	57.0	43.1	99.82	0.000
	Male	1,512	74.0	26.0		
**Age group*** **						
	18-39	683	64.8	35.2	16.47	0.000
	40-59	1,089	62.2	37.9		
	60+	1,408	69.3	30.8		
**Educational qualification****						
	Degree and above	1,413	67.9	32.1	9.57	0.020
	A level or equivalent	670	64.0	36.0		
	Below A level/equivalent	790	62.5	37.6		
	No qualification	312	63.1	36.9		
**Ethnic group*** **						
	White	2,878	66.7	33.3	40.01	0.000
	Asian/Asian British	166	55.3	44.7		
	Black/Black British	51	33.8	66.2		
	Other	64	60.1	39.9		
**Religion*** **	No religion	1,285	68.4	31.6	29.69	0.000
	Christian	1,600	65.5	34.5		
	Muslim	81	42.3	57.7		
	Other	227	54.4	45.6		

Factor	Category	Number	Supportive (%)	Unsupportive (%)	Chi-square value	P-value
**Thought of organ donation and decision registered on the Organ Donor register*** **						
	Yes, registered opt-in	1,388	72.8	27.2	76.65	0.000
	Yes, registered opt-out	51	54.3	45.7		
	Yes, cannot remember decision	49	69.2	30.8		
	Thought of but not registered	608	61.7	38.3		
	Not thought of donating	769	52.5	47.5		
**Were you aware of the possibility of transplanting animal organs into humans before the survey*** **						
	Yes	2,144	74.9	25.1	228.45	0.000
	No	1,048	47.2	52.9		
**If you needed an organ transplant, if any, would you be willing to accept one from a pig*** **						
	Yes	1,908	90.9	9.1	1400.00	0.000
	No	1,238	27.7	72.3		
**How will the availability of pig organ transplantation on the NHS affect your willingness to donate your organs after death?*** **						
	It would reduce my willingness to donate my organs	153	36.9	63.1		
	It would increase my willingness to donate my organs	218	42.8	57.2	104.41	0.000
	It would not affect my willingness to donate my organs	2,800	68.9	31.2		
**Region**						
	North East	146	62.5	37.5	6.40	0.845
	North West	333	64.0	36.1		
	Yorkshire and The Humber	273	67.2	32.8		
	East Midlands	240	65.2	34.8		

Factor	Category	Number	Supportive (%)	Unsupportive (%)	Chi-square value	P-value		Factor	Category	Number	Supportive (%)	Unsupportive (%)	Chi-square value	P-value
**Meat eating classification*** **									West Midlands	273	61.7	38.4		
	Vegetarian	138	46.4	53.6	101.66	0.000			East of England	309	68.3	31.7		
	Pescatarian	71	39.8	60.2					London	361	63.6	36.4		
	Vegan	43	26.3	73.7					South East	435	67.5	32.5		
	Mainly vegetarian but occasionally eat meat	289	59.5	40.5					South West	300	66.2	33.8		
	Eats meat, excluding pork	83	49.9	50.1					Wales	144	64.9	35.1		
	Eats meat	2,184	69.6	30.4					Scotland	253	63.5	36.5		
	None of these	383	65.0	35.0					Northern Ireland	126	67.9	32.1		
**Disability*** **								**Do you/ a family member/close friend, have any experience of organ donation?**						
	Yes, affects day-to-day life a lot	362	57.0	43.0	12.212	0.000			Yes	413	67.8	32.2	0.8206	0.365
	Yes, affects day-to-day life a little	562	65.3	34.7					No	2,778	65.0	35.1		
	Yes, does not affect day-to-day life	267	71.9	28.1										
	No disability	1,994	65.8	34.2										

Pearson's chi-squared (χ2) test p-value: ***p<0.01; **p<0.05. Row percentages are presented in the table.

Numbers responding to individual questions vary since respondents could choose not to answer questions.
